# Three-Dimensional Filial Piety Scale: Development and Validation of Filial Piety Among Chinese Working Adults

**DOI:** 10.3389/fpsyg.2019.02040

**Published:** 2019-09-06

**Authors:** Juan Shi, Fengyan Wang

**Affiliations:** ^1^Institute of Moral Education, Nanjing Normal University, Nanjing, China; ^2^School of Psychology, Nanjing Normal University, Nanjing, China

**Keywords:** filial piety, working adults, balance of interests, good affection, family role norms, three-dimensional filial piety model, scale development

## Abstract

The aim of this investigation is to develop a three-dimensional filial piety scale and explore its psychometric properties. Two studies are conducted based on Wang’s three-dimensional filial piety model. Study 1 consists of a review of the current literature, in-depth interviews, and feedback from the target group and experts. An initial 36-item scale using a bipolar Likert 6-point rating scale is developed. Then exploratory factor analysis is conducted on working adults (*n* = 617) to explore the dimensions and final items, and a 15-item scale with three factors is obtained. Study 2 confirms the factor structure of the new three-factor scale obtained from Study 1 using a confirmatory factor analysis with sample 1 (*n* = 585). Next, the criterion validity is tested with sample 2 (*n* = 248) and test–retest reliability with sample 3 (*n* = 67). The results support the model on which this scale is based and show three dimensions of filial piety, namely the balance of interests, good affection, and family role norms. As a valid, reliable scale, the three-dimensional filial piety scale can therefore be used in the Chinese context to measure filial piety for working adults of different genders and ages and in different cohabitation situations.

## Introduction

Due to the accelerated development of an aging population in China, the problem of providing for the aged has increasingly become a hot topic of social concern. Consistent with the Chinese concept of homesickness, a family pension is irreplaceable for old-age care and also helps reduce the burden on the government pension system (Fu et al., 2016; Hu, 2017). As a result, it has always been the main pension mode in China, and is expected by the vast majority of the elderly (Huang et al., 2017; Liu and Hu, 2017). As a core concept of Confucianism, filial piety contains important ideas about the way children should treat the elderly, and it plays an important role in shaping intergenerational relationships by providing ethical support for the family pension (Yeh and Bedford, 2003; Lee and Kwok, 2005). Adult children, especially working adults, are the primary source of old age care for the elderly through a family pension. As an increasing number of the younger generation and leaving their parents to study or earn a living, the environment and conditions conducive to nurturing filial piety are increasingly absent (Yan, 2016). Young people are facing increasing pressure to buy houses, pay for child care, and have stressful workloads. These all contribute to increasing the difficulty of filial practices (Zeng and Zou, 2017). Under this social and family background, the concept of filial piety for adult children has also changed. Therefore, it is extremely important to accurately grasp the psychology of filial piety for contemporary adult children in China.

### Psychological Measurement of Filial Piety

Since the beginning of the psychological research on filial piety in the 1970s, standardized tools for measuring filial piety have emerged (Ho and Lee, 1974; Yang et al., 1989; Ho, 1994; Sung, 1995). Researchers initially defined and measured filial piety from a one-way perspective, believing that filial piety is an authoritarian relationship, requiring children to absolutely obey their parents’ wishes, repay their parents’ sacrifices, safeguard family honor, and be responsible for the continuation of ancestral lineage (Ho and Lee, 1974).

With advances in the research, the emotional factor of filial piety has increasingly attracted the attention of researchers (Yang et al., 1989; Sung, 1995). Yeh and Bedford (2003) integrated authority and emotionality and suggested a dual filial piety model (DFPM). The DFPM and the dual filial piety scale (DFPS), developed on the basis of this model, contain two dimensions: (a) reciprocal filial piety, a kind of voluntary support, care, and love for one’s parents, which is motivated by the good nature of human beings, and entails a more balanced, two-way parent–child relationship, and (b) authoritative filial piety, which is motivated by compliance to the norms of social roles and often involves passive submission and absolute obedience to authority and entails an asymmetric parent–child relationship (Yeh, 2003; Yeh and Bedford, 2003; Yeh et al., 2013). The DFPM has been the most important theory, and the DFPS has been the most widely used scale in current filial piety research thus far. Both of them emphasize authority, as well as parental indebtedness and unconditional repayment (Lum et al., 2015). However, a majority of people no longer regard filial piety as an authoritative obligation in the 21st century (Chow, 2006; Lum et al., 2015), but rather as an intergenerational exchange of care needs and care capacities in an egalitarian parent-child relationship (Lee and Kwok, 2005; Lum et al., 2015). People see this as a way to establish a compromised commitment to care. In this view, children’s filial piety toward their parents should be based on their own abilities and resources (Whyte, 2004; Zhan, 2004; Lam, 2006; Lum et al., 2015). Therefore, the DFPM has limitations in reflecting the characteristics of contemporary filial piety.

The disappearance of obedience in the parent–child relationship effectively redefines the norm of filial piety (Yan, 2016). Lum et al. (2015) developed a new scale, the contemporary filial piety scale (CFPS). The CFPS not only suggests a paradigm shift from an authoritarian to an egalitarian parent–child relationship in contemporary filial piety, but also highlights that filial caregiving should be based on the abilities and resources of the offspring (Lum et al., 2015). These points reflect the modernity of CFPS. This scale contains two dimensions, namely, compassionate reverence and pragmatic obligations. The former is emotional caregiving and reasonable pursuit of parental aspirations, which is achieved through the sharing of life experiences and wisdom and is not based on unquestionable honor and glorification (Lum et al., 2015). The latter is a form of practical caregiving and is achieved through open exchanges of care needs and care capacities for establishing a compromised commitment to care (Lum et al., 2015). Lum et al. (2015) argued that this scale was based on “highlighting the conditional and utilitarian nuance of caregiving practices based on one’s abilities and resources.” The items in this scale, such as “provide financial subsistence to parents when they can no longer financially support themselves” and “arrange appropriate treatment for parents when they fall ill” underscore the conditionality of family care. However, the conditionality here is from the perspective of parents, referring to the conditions of parents’ need, such as, “when they can no longer financially support themselves” and “when they fall ill.” In other words, these items lack direct evaluation based on children’s own abilities and resources. Children are the main people practicing filial piety. Their own abilities and resources are important factors of affecting the filial mind and behavior (Whyte, 2004; Zhan, 2004; Lam, 2006; Lum et al., 2015). To better grasp the connotation of contemporary filial piety, the psychological characteristics of filial piety based on children’s own resources and abilities should be examined directly.

The DFPS, CFPS, and other scales, such as the filial behavior scale (Chen et al., 2007) and the filial expectation scale (Wang et al., 2010), are all based on the interactions between parents and children (Yeh and Bedford, 2003). However, filial piety is not simply the parent–child relationship. Adult children who bear the main duty of filial piety usually have the multiple roles of child, husband or wife, and parent in the family. Their family resources are allocated among three generations, rather than two generations (Di and Zheng, 2016). In addition, adult children also play a number of social roles (e.g., employee or leader). According to role conflict theory (Greenhaus and Beutell, 1985), it is difficult for people to meet the different social expectations of different interaction objects at the same time. In this way, the fulfillment of filial duty will inevitably affect the fulfillment of the obligations of another role. Therefore, when children face the choice of filial piety, they need to balance various roles and responsibilities, which is beyond the scope of the parent–child relationship.

### New Features of Filial Piety in Contemporary China

The development of internet technology has provided the younger generation with a more flexible and autonomous way of gaining employment. A constant updating of knowledge, skills, and information have replaced the life and work experience accumulated over long timescales and handed down from generation to generation (Zeng and Zou, 2017). As a result, the older generation’s status and power in the family have been gradually diminished. Under the background of this era, people continue to pursue equality, freedom, independence, autonomy, dignity, and happiness and renew their concepts of filial piety. Generally speaking, contemporary filial piety has three characteristics:

First, normativity still exists in filial piety. The decline of authoritative filial piety does not mean the decline and extinction of filial piety, but rather the transformation of the filial piety paradigm and its adaptability in response to social development. The value of filial piety as a moral norm still exists. The aggravation of aging, the absence of a social welfare system, and the persistence of the interdependent parent–child relationship mean that the family obligations of children will still play an important role in the personal welfare of parents for a long time to come (Qi, 2015). Therefore, filial piety, as a family ethic based on an egalitarian parent–child relationship, is still widely advocated by the society and has a binding effect on children (Sun, 2017).

Second, emotionality in filial piety is emphasized. The basic feeling of filial piety is based on the good nature of human beings and comes from gratitude for parenting and the daily interaction with parents (Yeh, 2009). The emotional nature of filial piety has not declined during the period of social transformation. People still strongly identify with the traditional filial piety that shows one’s true feelings to one’s parents, for example, “respecting parents” and “caring for parents” (Wang et al., 2014). Meanwhile, an egalitarian parent–child relationship has also been valued by children. People have begun to attach importance to two-way ideological and emotional exchanges between parents and children under an equal intergenerational relationship (Yan, 2016).

Third, rationality and autonomy in filial piety are strengthened. In contemporary egalitarian intergenerational relations, filial piety is increasingly regarded as an intergenerational exchange between upbringing from parents and support from children, with rationality and autonomy (Lee and Kwok, 2005; Lum et al., 2015). Children can be more rational based on their own ability and resources to choose the right way to show filial piety to their parents. Faced with the fact that living away from parents has become the normal social situation, children no longer stick to the old motto of “when parents are alive, children should not travel too far afield,” but regard “often bringing their spouse and children back home to visit their parents,” which was never a part of traditional filial piety, as one of the most important filial behaviors (Wang et al., 2014). Conversely, parents regard the “subcontracting filial piety” as an acceptable form of filial piety in which children hire family caregivers for parents or place them in nursing homes (Lan, 2002; Zhan et al., 2008; Luo and Zhan, 2012). In addition, more and more parents accept the fact that adult children may violate their parents’ wishes and regard “caring and supportive but not obedient” (*xiao er bu shun*) as a new understanding of filial piety (Yan, 2016). These characteristics not only reflect the increasing vitality and modernity of filial piety with the development of the times, but also conforms to the orthodox Confucian ideology of filial piety. As written in the *Classic of Family Reverence* (*Xiaojing*) in a section entitled *On Remonstrance* (*Jianzheng*):

If a father has a son who will remonstrate with him, he will not behave reprehensively (*buyi*). Thus, if confronted by reprehensible behavior on his father’s part, a son has no choice but to remonstrate with his father, and if confronted by reprehensible behavior on his ruler’s part, a minister has no choice but to remonstrate with his ruler. Hence, remonstrance is the only response to immorality. How could simply obeying the commands of one’s father be deemed filial? (Rosemont and Ames, 2009, pp. 113–114).

Therefore, the view that parents are always right and children should absolutely obey their parents is not the righteous meaning of good filial piety. The criterion in the section entitled *On the Way of Sons* (*Zidao*) by Xunzi is the righteousness of Confucian filial piety. It states that “you must carefully judge the manner of his ‘following’ before it can be described as ‘filial”’ (Knoblock, 1999, p. 949). Specifically, “to follow the dictates of the Way rather than those of one’s lord and to follow the requirements of morality rather than the wishes of one’s father constitute the highest standard of conduct” (Knoblock, 1999, p. 945). Obviously, the contemporary Chinese have realized the essence of filial piety.

### The Three-Dimensional Filial Piety Model

**FIGURE 1:**
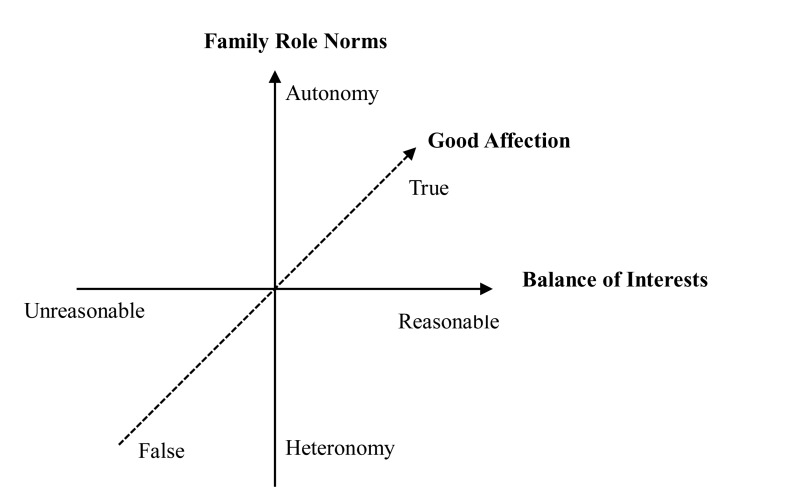
The three-dimensional filial piety model. Adapted from Wang and Zheng (2015). Copyright 2018 by F. Y. Wang (Reprinted with permission).

To make up for the deficiency of the DFPM and reveal the characteristics of contemporary filial piety more comprehensively, Wang and Zheng (2015, pp. 262–305) conducted a thorough analysis of the historical changes in the connotation of filial piety, classified filial piety systematically in terms of different levels, and then constructed a three-dimensional filial piety model (TDFPM; Figure 1). Each dimension of this model consists of two opposite poles: good affection (true–false), family role norms (autonomy–heteronomy), and balance of interests (reasonable–unreasonable). According to Wang and Zheng (2015, pp. 269–272), the definitions and contents of the three dimensions are as follows: *Good affection* refers to the emotions and feelings that children have for their parents, including true and false filial piety. The former refers to filial piety that embraces true feelings, and the latter refers to filial piety involving false hypocrisy, where one only wants to get some benefit from parents or to create the image of a “filial son/daughter” through the care and respect for parents. The fundamental difference between the two lies in the children’s motives: the former is for the purpose of treating parents in a kind and caring way, while the latter is strongly self-serving and instrumental. *Family role norms* refer to an individual’s behavior intention and reaction tendency to filial piety norms based on their own children’s roles. Children’s filial intention will be different when they abide by filial piety norms. Accordingly, filial piety can be divided into autonomous and heteronomous filial piety. The former refers to children who need external motivation to show filial piety to their parents. More succinctly, only after an individual perceives a tangible or intangible external pressure will they display recognition, emotion, and corresponding behavioral intentions or reaction propensity so as to fulfill their filial obligations. The latter refers to children who consciously show their filial attitude and filial behavior toward their parents. So, even when they are only guided by their conscience, children can have cognition, emotion, and corresponding behavioral intentions or reaction propensity so as to fulfill their filial obligations. *Balance of interests* refers to the balance of legitimate interests among offspring, parents, other family members, and society in the context of filial piety. Accordingly, filial piety can be divided into reasonable and unreasonable filial piety, depending on whether filial piety will infringe on the rights and interests of all persons concerned. Reasonable filial piety can be defined as moderate obedience of parents within one’s ability and without undermining one’s own or anyone else’s interests. On the premise of voluntariness, if children moderately sacrifice their personal interests to show filial piety to their parents, it is still regarded as reasonable filial piety. Unreasonable filial piety includes blind devotion to one’s parents (*yuxiao*) and one-way filial piety. The former refers to absolute obedience to the will of parents and sparing no effort to meet the demands of parents, while the latter refers to children making every effort to honor their parents regardless of how the parents treat them.

The TDFPM is an extension of the DFPM. It makes the division of each dimension more specific. In terms of the emotional aspect, the TDFPM mainly concerns whether the children’s emotions and feelings toward their parents are sincere or not. Previous studies on filial piety neglected motivation and considered filial emotion sincere by default (Yeh and Bedford, 2003; Yang, 2012; Lum et al., 2015). Motivation is regarded as the central process for the generation of both moral judgment and action (Kaplan, 2017). Individuals with high levels of affection for their parents may genuinely care for their fathers, or they may just want to benefit themselves. For example, after the death of the elderly, the children may hold a grand funeral in order to obtain economic benefits brought by the custom of “presenting money for a funeral” (*sui fen zi*) or the good reputation of a “filial son/daughter” (Li and Fang, 2018). Hence, in order to accurately measure children’s feelings toward their parents, it is necessary to consider children’s filial motivation.

The TDFPM replaces authoritative filial piety with the dimension of family role norms. This transformation corresponds to the regression of authoritative filial piety in contemporary society and preserves the role of filial piety as a cultural norm and moral standard. Under the trend of population aging, a great deal of government attention has been focused on elder care and filial practices (Bedford and Yeh, 2019). Therefore, the normativity of filial piety is conducive to the normal operation of elder care, especially the family pension.

By distinguishing reasonable filial piety from unreasonable filial piety, the model provides theoretical guidance for children to engage in reasonable filial piety on the basis of their abilities and resources. In this case, filial piety can operate within the framework of morality. As for children’s reasonable rights and interests, economic interests are an important part. Children’s multiple roles in the family and society mean that their economic income has many responsibilities, such as supporting their spouses and children. In this way, when they give financial support to parents, adult children need to balance multiple roles. Opportunity cost is another important part of children’s reasonable rights and interests in allocating parental responsibilities. The responsibility of caring for and accompanying parents is mainly borne by children with a relatively lower opportunity cost of caring (e.g., lower income, lower education) (Liu and Hu, 2017).

Furthermore, by increasing the dimension of balance of interests, the explanatory scope of the model is enlarged. Previous studies have found that the degree of filial cognition among college students is high (Cao and Yeh, 2014), while in China’s poorer areas, such as Li Village in Henan province and the immigrant village of Jingshan county in Hubei province, filial piety has collapsed; instead of fulfilling their obligation to support their parents, children have made negative claims about their parents (Chen, 2009; Li, 2014). The TDFPM can explain these differences as follows: College students, whose parents are mostly young and healthy, have not yet assumed the responsibility of supporting their parents and have not realized the difficulties of filial piety. Their parents usually do not need them to either contribute their time or money, or sacrifice their personal development to fulfill filial piety. Therefore, college students do not give up filial piety because of their limited abilities or protection of their own interests; thus, their level of filial piety is generally high. In contrast, children in poor families may love their parents deeply and be clearly aware of their responsibility and obligation to support their parents, but, due to the restrictions of economic strength and work, they cannot fulfill filial piety. In other words, even though they have both filial affection and intention, they do not fulfill filial piety due to their lack of personal ability and limited resources.

### The Present Research

In this study, the method of combining theory-driven and data-driven information was used. The TDFPM was the theoretical basis and working adults, the main practitioners of family care, were the participants in this study. Two sequential studies were involved in the current research with the aim to create a psychometrically valid measure of filial piety. Study 1 details the development of the item pool to measure filial piety in working adults, and the factor structure obtained by exploratory factor analysis (EFA). Study 2 examined the validity of the factors identified in Study 1 via confirmatory factor analysis (CFA). Then, the reliability and validity were also tested.

## Study 1: Scale Construction and Development

This study aimed to develop a new scale to measure filial piety in working adults. Theoretical analysis, preexisting scales analysis, and in-depth interviews were used comprehensively to develop a primary item pool. Then the primary item pool was evaluated by both the experts and the target investigation group, and this was the second item pool. The items in the second item pool were then subjected to EFA.

### Item Generation

**FIGURE 2:**
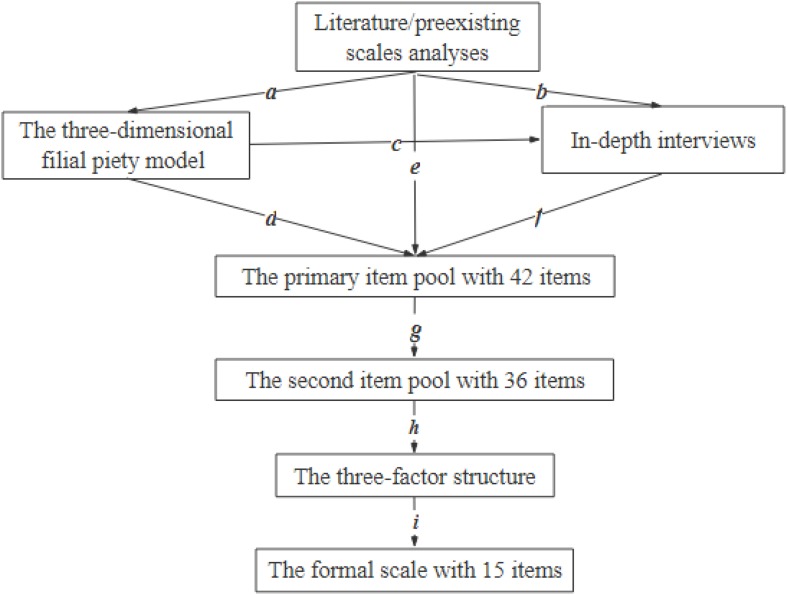
Process of generating initial 15 items of TDFPS. In this figure, “a” proposed the three-dimensional filial piety model based on literature and preexisting scales analyses; “b and c” formed in-depth interviews outlines based on literature and preexisting scale analyses and the three-dimensional filial piety model, respectively; “d, e, and f” generated items based on the model, the analyses of literature and preexisting scales and the in-depth interviews, respectively; “g” assessed these items by experts and the target group with low education; “h” conducted the EFA; “i” conducted the CFA to verify the three-factor structure.

To generate items that reflected the content of contemporary filial piety, the processes shown in Figure 2 were conducted. Based on the TDFPM and aforementioned literature, an in-depth interview outline was developed that, included the following questions: (1) “What do you think filial piety is? Please talk about your understanding of filial piety. You can explain it by giving examples of ‘filial person’ and ‘filial behavior.”’ (2) “Under what circumstances do you or the people around you usually act with filial piety?” (3) “What motivates you or the people around you to show filial piety in these circumstances? Subjective or objective reasons, or both?” Individual interviews were conducted with a convenience sample of ten participants. All of the participants were working people, with ages ranging from 31 to 52 (M = 39.1, SD = 7.16, five female). Among them, three interviewees lived with their parents in the same house; three interviewees lived in the same city as their parents but not in the same house; and the other four interviewees lived in different cities than their parents. The distribution of age, gender, and distance from parents of these 10 interviewees was similar to that of the samples used in the EFA. Each participant received 10 yuan (around 1.44 dollars) as a reward. The results of the in-depth interviews were categorized. For example, “Helping parents when they need it” was classified as “heteronomy filial piety”; “Visiting parents when I have time, without waiting for their request” was classified as “autonomy filial piety.”

A primary item pool was created using literature analyses, in-depth interviews, and related filial piety scales, such as the DFPS (Yeh and Bedford, 2003), the filial behavior scale (Chen et al., 2007), and the CFPS (Lum et al., 2015). These items were further refined by (a) deleting ambiguous items, (b) deleting items with high face validity, and (c) classifying items with similar meanings. Finally, a primary item pool with 42 items was obtained (see Supplementary Appendix).

Each of the 42 items contained two sentences with opposite meanings; one for autonomy, true, or reasonable filial piety, and the other one for heteronomy, false, or unreasonable filial piety. Participants were asked to choose one of the two statements they identified with more and then to mark the degree of conformity with the selected statement on a three-point scale (1 for slightly identify, 2 for moderately identify, 3 for completely identify). The options were recoded for statistical analysis. For those who chose the sentence that represented negative filial piety (i.e., heteronomy, unreasonable, and false filial piety), “completely identify” was recoded to 1, “moderately identify” was recorded to 2, and “slightly identify” was recoded to 3. For those who chose the sentence belonging to positive filial piety (i.e., autonomy, reasonable, or true filial piety), “completely identify” was recorded to 6, “moderately identify” was recorded to 5, and “slightly identify” was recorded to 4. In this way, each item was rated on a 6-point bipolar Likert scale. Take item 1 as an example, if participants thought that the first sentence “I take initiative to accompany my parents if time permits” rather than the second one “I passively accompany my parents only when they ask” was more consistent with their own situation, they should choose the first sentence. If they were moderately identified with the first sentence, they should choose 2 “moderately identify,” and the final score was reassigned to “5.” Compared with the unipolar Likert scale, the bipolar Likert scale can reduce the number of questions and makes it easier for subjects to cooperate with the test (Tzeng et al., 1991). The 42 items were divided into three dimensions, with 14 items for each dimension.

These 42 items were then further evaluated. First, an expert assessment was conducted. One professor and five doctoral students of psychology were invited to evaluate the content validity. This included (a) evaluating the consistency of each item with the operational definition of the subordinate dimension; that is, whether the item can accurately express the content defined by the dimension; (b) examining the accuracy, comprehensibility, and redundancy of each item; and (c) assessing the socially desirable response. According to the above evaluation, the items were discussed one by one, and items with duplicate content, unclear expression, obvious inconsistency with the operational definition, and highly socially desirable response were deleted. Second, the items were assessed by a target group with a low education. Eleven people (five males, six females) aged 35–60 years (M = 48.91, SD = 7.49) were selected for one-on-one testing using convenience sampling. All of them had less than an undergraduate degree. Every time the subjects completed a question, they reported their understanding of the question to the investigator (the first author of this paper) and gave feedback on whether the item was ambiguous or inappropriate. According to the feedback of these 11 participants, the expression of the items was modified. After assessment by experts and the target group, six items were deleted, and the second item pool was eventually formed, which contained 36 items. All of these 36 items were rated on a 6-point bipolar Likert scale. Among them, 21 items listed positive filial piety first and negative filial piety later, and the other 15 items were the opposite.

### Participants and Procedure

Scales, including demographic items and the second item pool with 36 items, were published using the professional online platform “*Wenjuanxing.*” At the beginning of the survey, participants needed to read the informed consent and make a choice between “read, agree” and “read, disagree.” Only those who chose the former one could continue to complete the following questionnaire.

A sample of 672 working adults completed the 36-item scale. Participants were excluded for missing data or providing obviously repetitive answers (*n* = 55, 8.18%). Thus, 617 valid respondents, aged 20–66 (M = 33.29, SD = 6.97) (Table 1), were included in the following statistical analysis.

**TABLE 1:** Demographic information of participants.

				**Study 2**					
		**Study 1**		**Sample 1**		**Sample 2**		**Sample 3**	
		**(*n* = 617)**		**(*n* = 585)**		**(*n* = 231)**		**(*n* = 67)**	
		***n***	**%**	***n***	**%**	***n***	**%**	***n***	**%**
Gender	Male	277	44.9	239	40.9	90	39.0	29	43.3
	Female	340	55.1	346	59.1	141	61.0	38	56.7
Age	≤30	251	40.7	248	42.4	105	45.5	31	46.3
	31–40	276	44.7	223	38.1	68	29.4	33	79.3
	≥41	90	14.5	114	19.5	58	25.1	3	4.5
Marital status	Unmarried	124	20.1	138	23.6	60	26.0	10	14.9
	Married	485	78.6	438	74.9	168	72.7	55	82.1
	Divorced and windowed	8	1.3	9	1.5	3	1.3	2	3.0
Living area	Urban	341	55.3	318	54.4	150	64.9	31	46.3
	Rural	276	44.7	267	45.6	81	35.1	36	53.7
Educational level	High school and below	152	24.6	122	20.9	24	10.4	14	20.9
	Undergraduate	294	47.6	290	49.6	128	55.4	31	46.3
	Graduate	171	27.7	173	29.6	79	34.2	22	32.8
Fertility condition	With child/children	418	67.7	391	66.8	144	62.3	50	74.6
	Childless	199	32.3	194	33.2	87	37.7	17	25.4
Annual household income	<100,000 RMB	223	36.1	222	37.9	74	32.0	24	35.8
	100,000–200,000 RMB	258	41.8	222	37.9	86	37.2	20	29.9
	>200,000 RMB	136	22.0	141	24.1	71	30.7	23	34.3
Parents’ pension	Both parents have	140	22.7	144	24.6	71	30.7	13	19.4
	One of the parents has	93	15.1	116	19.8	53	22.9	9	13.4
	Neither parent has	384	62.2	325	55.6	107	46.3	45	67.2
Cohabitation forms	Same house	173	28.0	153	26.2	65	28.1	22	32.8
	Same city/town, different house	201	32.6	193	33.0	69	29.9	19	28.4
	Different city/town	243	39.4	239	40.9	97	42.0	26	38.8

### Statistical Analysis

First, the critical ratio (CR) and item-total correlation was used for item analysis to test whether these items had enough discrimination and were consistent with the scale. Items with low CR (*t* < 3.00, *p* < 0.01) were deleted, as they may have low discrimination, and items with an item–total score correlation less than 0.3 were also dropped, as they may inconsistent with the entire construct (Wu, 2013).

Second, the factorability was assessed using the Bartlett’s test of sphericity, the Kaiser–Meyer–Olkin (KMO) test, and the measures of sampling adequacy (MSA). Items were considered appropriate for factor analysis when the result of Bartlett’s test was statistically significant, and the KMO and MSA value was 0.80 or higher (Kaiser, 1974).

Third, the principal components analysis and varimax rotation were used to explore the latent structure of the scale. The criteria for factors and item reduction were as follows: (a) eigenvalues greater than 1 and the scree plot were used to determine the number of factors; (b) the factor that contained less than three items was dropped; (c) items with a secondary factor loading of 0.30 or higher were defined as cross-items and dropped; (d) items that loaded at ≥0.50 and with a communality value ≥0.4 were finally retained (Bosworth et al., 1999; Worthington and Whittaker, 2006). Factorial simplicity was evaluated using the index of factorial simplicity (IFS) and the scale fit index (SFI). Items with IFS ≥ 0.80 were considered meritorious, and those with IFS ≥ 0.60 were considered mediocre (Kaiser, 1974). Items with SFI ≥ 0.80 were considered desirable (Fleming, 2003).

These analyses were conducted using SPSS 21.0.

### Results and Discussion

Based on the item analysis of the 36 items, the CR of all items was applicable, and four items were removed due to their item–total score correlation being lower than 0.30. The remaining 32 items were moved to the next analysis.

An EFA was conducted on the 32 items to determine the underlying factor structure of the items. The KMO value (=0.903) and Bartlett’s test of sphericity (*p* < 0.001), indicated that these items were adequate for factor analysis. Then, according to the aforementioned criteria (a) through (d), the items that did not meet the requirements were deleted. Finally, 15 items with strong loadings onto three factors without cross-loading were retained (Table 2). Both the eigenvalues and the scree plot suggested a three-factor solution, which explained 55.83% of the variance. All of the individual IFS values were above 0.60, and the SFI values of the three factors were above 0.8, indicating desirable factorial simplicity (Table 2).

**TABLE 2:** Three-dimensional filial piety scale exploratory factor analysis (*n* = 617).

**Item**	**M (SD)**	**GA**	**FRN**	**BI**	***h*^2^**	**IFS**
34	5.49 (0.69)	0.825			0.723	0.914
27	5.45 (0.71)	0.810			0.728	0.854
16	5.53 (0.62)	0.764			0.679	0.789
29	5.39 (0.72)	0.752			0.643	0.818
36	5.39 (0.73)	0.699			0.653	0.634
1	4.09 (1.34)		0.731		0.535	0.999
35	4.92 (1.36)		0.697		0.562	0.802
9	4.92 (1.32)		0.677		0.533	0.798
24	4.90 (1.30)		0.644		0.548	0.659
14	4.87 (1.24)		0.626		0.465	0.765
13	4.90 (1.19)			0.697	0.488	0.994
18	4.96 (1.16)			0.628	0.426	0.891
11	4.69 (1.30)			0.623	0.423	0.881
15	5.12 (0.81)			0.622	0.499	0.681
32	5.21 (0.92)			0.600	0.470	0.653
Dimension total		27.25	23.70	24.88		
Eigenvalues		5.427	1.717	1.231		
Percent variance explained (%)		36.179	11.448	8.204		
SFI		0.853	0.859	0.905		

Following an appropriate process, Study 1 resulted in a 15-item scale with three factors. This model was consistent with the TDFPM. Based on this model, the 15-item scale was named the Three-Dimensional Filial Piety Scale (TDFPS). Accordingly, these three factors were named good affection (GA, five items), family role norms (FRN, five items), and balance of interests (BI, five items). However, this three-factor structure of filial piety was based on one sample. Therefore, in Study 2, a confirmatory factor analysis and validity assessment were conducted in a replication sample.

## Study 2: Confirmatory Factory Analysis and Validity Assessment

The purpose of Study 2 was to replicate the three-factor structure and test the validity and reliability of the 15-item TDFPS using new samples. A CFA was conducted to test the three-factor model of filial piety. The internal consistency reliability, test–retest reliability, structural validity, criterion validity, and convergent validity were also conducted to test the reliability and validity of the new scale.

### Participants and Procedure

Measurement tools, including demographic items, the TDFPS, and scales used for criterion validity, were published using the professional online platform “*Wenjuanxing.*” The measurement process was the same as that of Study 1. Three samples were collected, of which the latter two were followed up from the first one. Their demographic information is shown in Table 1.

Sample 1: A total of 649 adults completed the 15-item TDFPS. Participants were excluded for missing data or for providing obviously repetitive answers (*n* = 64, 9.86%). Thus, 585 valid respondents, aged 20–62 (M = 34, SD = 8), were included in the CFA, reliability analysis, and validity analysis. Due to the need for repeated tests, participants were informed at the end of the scale that the same survey would be conducted 1 month later. Participants willing to continue to participate in the study were invited to leave their mobile phone number or WeChat account.

Sample 2: In sample 1, 248 participants completed the DFPS and CFPS while completing TDFPS. These 248 participants were excluded for missing data or for providing obviously repetitive answers (*n* = 17, 6.85%). Thus, 231 valid respondents, aged 20–60 (M = 34, SD = 8.6), were included in the criterion validity analysis.

Sample 3: 4 weeks after the survey of sample 1, the TDFPS was used to measure the 72 participants who belonged to sample 1 and were willing to participate in repeated measurements and leave their contact information. Five participants (6.94%) were excluded for missing data or for providing obviously repetitive answers. Thus, 67 valid respondents, aged 20–54 (M = 34, SD = 8.6), were included in the test–retest reliability analysis.

### Measures

#### Three-Dimensional Filial Piety Scale (TDFPS)

The 15-item TDFPS we developed was used. Participants were asked to rate the extent of their agreement with each item using a 6-point bipolar Likert scale.

#### Dual Filial Piety Scale (DFPS)

Developed by Yeh and Bedford (2003), the 16-item DFPS is rated on a 5-point Likert scale ranging (1 = definitely disagree, and 5 = definitely agree). The scale contains eight items on reciprocal filial piety (RFP) and eight items on authoritarian filial piety (AFP). Several studies have supported the value and validity of the DFPS (Leung et al., 2010; Jin et al., 2011; Chen and Ho, 2012). In the current study, the Cronbach’s alpha coefficient of DFPS was 0.711.

#### Contemporary Filial Piety Scale

Developed by Lum et al. (2015), the 10-item CFPS has strong psychometric properties and can assess contemporary filial piety in a simple and efficient way. The scale contains six items on pragmatic obligation (PO) and four items on compassionate reverence (CR). The CFPS employs a 5-point Likert scale (1 = very unimportant, 5 = very important). The scale’s Cronbach’s alpha coefficient was 0.88, and the CFI was 0.95, indicating high goodness of fit (Lum et al., 2015). In the current study, the Cronbach’s alpha coefficient was 0.758.

### Statistical Analysis

First, the factor structure examination of TDFPS was conducted by CFA using Mplus 7.0 with sample 1, a new and independent sample. Model fit was examined using the following indicators: (a) the normed χ^2^, with a value of <2 considered “very good” (Schreiber et al., 2006) and 2–5 considered “acceptable” (Wu, 2013); (b) the standardized root mean square residual (SRMR), with a value of 0.08 or less indicative of a good fit (Hu and Bentler, 1999); (c) the root mean square error of approximation (RMSEA), with a value of 0.05 or less considered a “good fit” and 0.05-−0.08 a “reasonable fit” (MacCallum et al., 1996); (d) the comparative fit index (CFI), with a value of 0.90 or more considered as “good” (Hu and Bentler, 1999); and (e) the Tucker-Lewis Index (TLI), with a value of 0.90 or more considered great. A measurement invariance across gender, age, and cohabitation situation was conducted. In order to be concise and avoid confusion, the conventions set forth by Steenkamp and Baumgartner (1998), including configural, metric, and scalar, were used. The CFI and RMSEA change values of 0.01 or less were considered acceptable (Cheung and Rensvold, 2002).

Second, the reliability of the new scale was tested using the internal consistency reliability and test–retest reliability on SPSS 21.0. The internal consistencies reliability of items, dimensions, and total scale were tested, respectively using *R*^2^, composite reliability (CR), Cronbach’s alpha coefficient, and split-half reliability on sample 1. Values of CR, Cronbach’s alpha coefficient, and the split-half reliability higher than 0.7 are indicative of an acceptable fit, with values higher than 0.8 suggesting excellent or good fit (Hair et al., 2006; Wu, 2013). The test–retest reliability was tested on sample 3 and examined via a two-tailed Pearson correlation.

Third, the validity of the new scale was conducted using structural validity, criterion validity, and convergent validity on SPSS 21.0. In addition to the CFA, the structural validity was further conducted using correlations between each item and its corresponding dimension and among different dimensions, as well as between each dimension and the total scale on sample 1. The DFPS and CFPS were used to calculate the criterion validity on sample 2. Both the DFPS and the CFPS are effective tools for measuring filial piety, but they differ from the TDFPS in structure. Therefore, the TDFPS was hypothesized to have low to medium positive associations with the DFPS and CFPS. The GA emphasized the emotional elements and good motives, the FRN emphasized the consciousness and initiative, and the BI emphasized rationality and morality. These are all consistent with RFP, PO, and CR to a certain extent. Accordingly, it was assumed that GA, FRN, and BI would be positively correlated with RFP, PO, and CR. In addition, the BI emphasizes filial piety within a reasonable scope, which is in conflict with AFP that advocates absolute obedience. Therefore, the BI was hypothesized to have a negative correlation with the AFP. The average variance extracted (AVE) was used to evaluate the convergent validity on sample 1. A value of 0.50 or more was considered as “accepted” (Fornell and Larcker, 1981).

### Results and Discussion

#### Confirmatory Factor Analysis of the TDFPS

**FIGURE 3:**
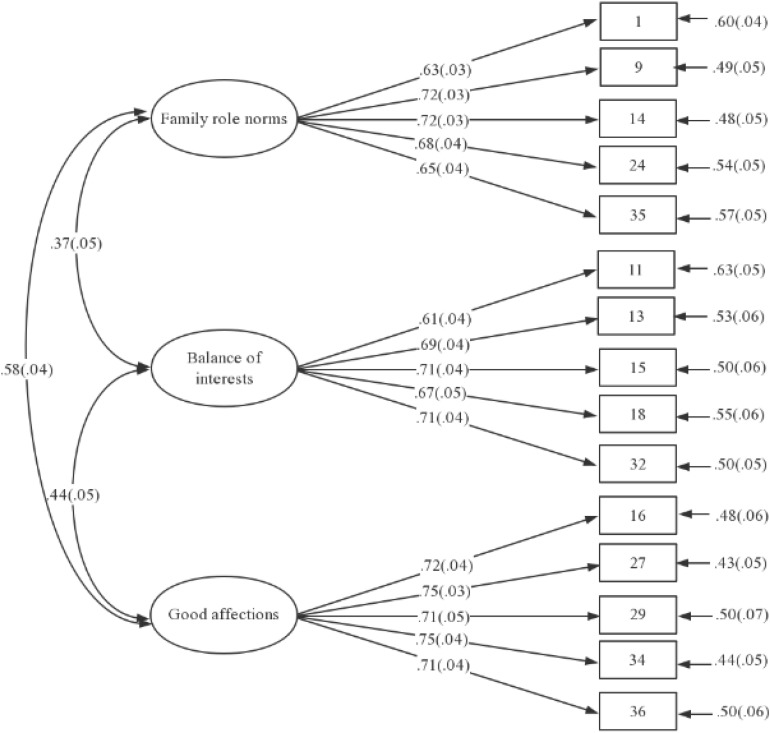
Confirmatory factor analysis results on TFPS.

A CFA was conducted to verify the three-factor structure of the TDFPS identified in the EFA. The absolute values of the skewness coefficients ranged from 0.640 (item 1) to 2.334 (item 29) (>2), and the absolute values of the kurtosis coefficients ranged from 0.206 (item 11) to 9.351 (item 29) (>7) (Table 3). Therefore, the MLM estimation, which is robust and suitable for estimating the parameters of a skewed distribution (Wang, 2014, p. 97), was used in this study. Both the one-factor model, which refers to a simple primary model in which all 15 items were affected by the same latent variable, and the three-factor model which was developed based on the EFA results, were tested. The goodness-of-fit indices of the two models (Table 4) showed that the three-factor model fit better than the one-factor model. In addition, in the three-factor model, the normed χ^2^ (=2.275 < 5) was acceptable, the SRMR (=0.048 < 0.08), RMSEA (=0.047 < 0.05), CFI (=0.955 > 0.90), and TLI (=0.946 > 0.90) also fit well. Consequently, the three-factor model was finally accepted. All of the standardized factor loadings of the three-factor model were statistically significant (*p* < 0.001), and all of the items significantly loaded onto the same factor in the CFA as they had in the EFA (Figure 3).

**TABLE 3:** Three-dimensional filial piety scale descriptive and correlation information of CFA (*n* = 585).

					**Total**			
**Factor**	**Item**	**M (SD)**	**Skew**	**Kurtosis**	**scale**	**FRN**	**BI**	**GA**
FRN					0.82^∗∗^	1		
	1	4.32 (1.23)	–0.64	–0.48	0.74^∗∗^			
	9	4.99 (1.26)	–1.37	0.91	0.78^∗∗^			
	14	5.01 (1.14)	–1.36	1.31	0.76^∗∗^			
	24	4.94 (1.23)	–1.35	1.06	0.75^∗∗^			
	35	4.90 (1.27)	–1.21	0.51	0.75^∗∗^			
BI					0.71^∗∗^	0.28^∗∗^	1	
	11	4.77 (1.13)	–0.94	0.21	0.74^∗∗^			
	13	4.91 (1.01)	–1.28	1.47	0.77^∗∗^			
	15	5.00 (0.86)	–1.19	2.54	0.74^∗∗^			
	18	4.88 (1.05)	–1.20	1.31	0.76^∗∗^			
	32	5.21 (0.96)	–1.67	3.34	0.75^∗∗^			
GA					0.73^∗∗^	0.47^∗∗^	0.35^∗∗^	1
	16	5.60 (0.61)	–1.88	7.01	0.78^∗∗^			
	27	5.51 (0.67)	–1.98	8.35	0.80^∗∗^			
	29	5.49 (0.73)	–2.33	9.35	0.80^∗∗^			
	34	5.55 (0.66)	–2.11	8.20	0.80^∗∗^			
	36	5.43 (0.73)	–2.09	7.87	0.78^∗∗^			

*^∗^*p* < 0.05, ^∗∗^*p* < 0.01*

**TABLE 4:** Fitting indices of models (*n* = 585).

	**χ^2^**	**df**	**χ^2^/df**	**TLI**	**CFI**	**AIC**	**BIC**	**SRMR**	**RMSEA(90% CI)**
One-factor	942.567^∗^	90	10.47	0.595	0.653	21907.129	22103.851	0.112	0.127 (0.120, 0.135)
Three-factor	197.955^∗^	87	2.275	0.946	0.955	20937.430	21147.267	0.048	0.047 (0.038, 0.055)

*^∗^*p* < 0.05.*

Measurement invariance provides empirical support for researcher decisions to interpret between-group comparisons as differences in degree rather than differences in kind (Geldhof and Stawski, 2015). A series of progressively more stringent multiple-group CFAs were run to test the measurement invariance across gender, age, and cohabitation situations. The measurement invariance analyses conducted in the current study included three levels: (a) configural invariance: provides the baseline value against which all subsequently specified equivalence models were compared and to test the equivalence of the factor structure across groups; (b) metric invariance: a stronger test of factorial invariance than the configure invariance. It analyzes the equivalence of the factor loadings across groups; and (c) scalar invariance: examines the equivalence of the factor loadings and item intercepts across groups (Vandenberg and Lance, 2000; Rensvold and Cheung, 2001; Byrne, 2008).

The results of the gender invariance analyses showed that the configural invariance across gender was supported (CFI = 0.953, TLI = 0.943, RMSEA = 0.048, and SRMR = 0.055) (Table 5). Against configural invariance, the subsequently specified equivalence models were further compared. Different values were found between the metric and configural invariance (ΔCFI = 0.002, ΔRMSEA = −0.002), indicating that the metric invariance was supported. Change values between the scalar and metric invariance (ΔCFI = −0.001, ΔRMSEA = −0.001) supported scalar invariance. These results show that the three-factor structure of the TDFPS does measure the same construct in men and women.

**TABLE 5:** Testing measurement invariance of gender, age, and cohabitation form (*n* = 585).

**Model**	**Model fit**						**Model comparison**		
	**S-Bχ^2^**	**Df**	**CFI**	**TLI**	**RMSEA**	**SRMR**	**Models**	**ΔCFI**	**ΔRMSEA**
**Gender invariance**									
M1: configural invariance	292.527^∗^	174	0.953	0.943	0.048 (0.038, 0.058)	0.055			
M2: metric invariance	299.741^∗^	186	0.955	0.949	0.046 (0.036, 0.055)	0.057	M2:M1	0.002	−0.002
M3: scalar invariance	313.912^∗^	198	0.954	0.951	0.045 (0.035, 0.054)	0.058	M3:M2	−0.001	−0.001
**Age invariance**									
M1: configural invariance	407.959^∗^	261	0.947	0.936	0.054 (0.043, 0.064)	0.059			
M2: metric invariance	441.028^∗^	285	0.943	0.937	0.053 (0.043, 0.062)	0.068	M2:M1	−0.004	−0.001
M3: scalar invariance	476.524^∗^	309	0.939	0.938	0.053 (0.043, 0.062)	0.069	M3:M2	−0.004	−0.001
**Cohabitation form invariance**									
M1: configural invariance	409.826^∗^	261	0.944	0.933	0.054 (0.044, 0.064)	0.061			
M2: metric invariance	441.770^∗^	285	0.941	0.935	0.053 (0.043, 0.063)	0.073	M2:M1	−0.003	−0.001
M3: scalar invariance	473.165^∗^	309	0.939	0.937	0.052 (0.043, 0.061)	0.074	M3:M2	−0.002	−0.001

*^∗^*p* < 0.05.*

Age invariance was measured using the same method. Participants were divided into three groups according to age: age_group 1_ < 31 (*n* = 248, M = 27.64, SD = 2.28), 31 ≤ age_group 2_ < 40 (*n* = 223, M = 34.19, SD = 2.84), and age_group 3_ ≥ 40 (*n* = 114, M = 47.45, SD = 4.27). The results showed that the configural invariance (CFI = 0.947, TLI = 0.936, RMSEA = 0.054, and SRMR = 0.059) was supported (Table 5). Furthermore, the different values between the metric and configural invariance model (ΔCFI = −0.004, ΔRMSEA = −0.001), as well as different values between the scalar and metric invariance (ΔCFI = −0.004, ΔRMSEA = −0.001), separately indicated the acceptable metric invariance and scalar invariance. These findings indicated that the three-factor structure of TDFPS does measure the same construct in different age groups.

The invariance was then measured across cohabitation forms (live in the same house with parents, live in the same city/town but in a different house than parents, and live in a different city/town than parents). The configural invariance (CFI = 0.944, TLI = 0.933, RMSEA = 0.054, and SRMR = 0.061) was supported (Table 5). Different values between the metric and configural invariance model (ΔCFI = −0.003, ΔRMSEA = −0.001) and different values between the scalar and metric invariance (ΔCFI = −0.002, ΔRMSEA = −0.001) separately demonstrated that the metric invariance and scalar invariance were supported. These findings indicated that the TDFPS does measure the same construct in different cohabitation situations.

#### Reliability of the TDFPS

##### Internal consistency reliability

The internal consistency reliability was then tested (Table 6). The *R*^2^ of each item ranged from 0.370 to 0.569, which was greater than 0.25, reaching a good level. The CR of each dimension was above 0.8, an acceptable level. These results showed that the intrinsic quality of the model was good. The Cronbach’s alpha coefficient of the total scale was 0.85, and that of each dimension exceeded 0.8. The split-half reliability of the total scale was 0.75, and that of each dimension exceeded 0.8. The Cronbach’s alpha and the split-half reliability met high and very high measurement standards, respectively. The *R*^2^, Cronbach’s alpha coefficient, and split-half reliability results showed that the three-dimensional scale of the filial piety had good cross-item stability.

**TABLE 6:** Psychometric properties of the TDFPS (*n* = 585).

**Factor**	***R*^2^**	**CR**	**AVE**	**Cronbach’s α**	**Spearman–Brown**	**Test–retest reliability**
FRN	0.398∼0.519	0.812	0.465	0.812	0.805	0.907^∗∗^
BI	0.370∼0.500	0.808	0.458	0.804	0.802	0.844^∗∗^
GA	0.500∼0.569	0.850	0.530	0.847	0.859	0.816^∗∗^
Total scale				0.848	0.752	0.900^∗∗^

*^∗∗^*p* < 0.01.*

##### Test–retest reliability

The results of 4-week test–retest reliability showed that the values of both the scale (*r* = 0.900, *p* < 0.01) and the three dimensions (*r*_frn_ = 0.907, *p* < 0.01; *r*_bi_ = 0.844, *p* < 0.01; *r*_ga_ = 0.816, *p* < 0.01) were very good (Table 6). These results indicated that the scale had good cross-time stability.

#### Validity of the TDFPS

##### Structural validity

The three-factor structure identified in the EAF was verified using CFA. The CFA results showed statistically significant standardized factor loadings (*p* < 0.001) (Figure 3) and acceptable (the normed χ^2^) and even great (SRMR, RMSEA, CFI, and TLI), goodness-of-fit indices (Table 4). Then, the correlations between each item and its corresponding dimension, among different dimensions, as well as between each dimension and the total scale were tested successively (see Table 3). The results were as follows: (a) a moderate to high positive correlation between each item and the corresponding dimension (0.74 ≤ *r* ≤ 0.80, *p*s < 0.01), indicating that the concept of each item was consistent with that of the corresponding dimension; (b) a moderate to low positive correlation among the dimensions (*p*s < 0.01, 0.28 ≤ *r* ≤ 0.47), indicating that the factors represented by the different dimensions were not only in the same direction, but also different and could not be substituted for each other; (c) highly positive correlations between the dimensions and the total score (*p*s < 0.01, 0.71 ≤ *r* ≤ 0.82), indicating that the dimensions were consistent with the concept of the total scale. These results demonstrated that the three-factor structure was both independent and interrelated, and had great consistency with the entire scale. Therefore, the three-factor theoretical structure of the TDFPS was strong.

##### Criterion validity

The DFPS and CFPS were used to calculate the TDFPS’s criterion validity. First, the common method biases were examined using Harman’s single-factor test. The results showed that the eigenvalues of 11 factors were greater than 1 without rotation, and the variance explained by the first factor was 19.55%, which was less than the critical standard of 40% (Podsakoff et al., 2003; Huang et al., 2016). This indicated that the deviations of the common methods in this study were not obvious. Then correlation analyses were conducted, and the results showed that the TDFPS had strong criterion validity (Table 7). The TDFPS scores were modestly positively correlated with the DFPS scores (*r* = 0.14, *p* < 0.05) and the CFPS scores (*r* = 0.34, *p* < 0.01). Specifically, FRN, BI, and GA had modest to moderate positive correlations with RFP, PO, and CR (*p*s < 0.05). In addition, the AFP was modestly negatively correlated with the BI (*p* < 0.01) and had no correlation with the FRN or GA (*p*s > 0.05).

**TABLE 7:** Correlations of TDFPS with DFPS and CFPS (*n* = 231).

**TDFPS**	**DFPS**			**CFPS**		
	**RFP**	**AFP**	**Total**	**PO**	**CR**	**Total**
FRN	0.314^∗∗^	–0.032	0.139^∗^	0.200^∗∗^	0.292^∗∗^	0.303^∗∗^
BI	0.243^∗∗^	–0.198^∗∗^	–0.036	0.147^∗^	0.167^∗^	0.188^∗∗^
GA	0.441^∗∗^	0.008	0.239^∗∗^	0.355^∗∗^	0.197^∗∗^	0.300^∗∗^
Total	0.418^∗∗^	–0.097	0.140^∗^	0.287^∗∗^	0.295^∗∗^	0.344^∗∗^

*^∗^*p* < 0.05, ^∗∗^*p* < 0.01.*

##### Convergent validity

The AVE was conducted as a measure of the convergent validity. In the current study, the AVE ranged from 0.458 to 0.530 (Table 6) and was greater than the recommended level of 0.5. This indicated adequate levels of convergent validity (Fornell and Larcker, 1981).

## General Discussion

### Reliability and Validity

The aim of the present study was to develop and validate a new measure to assess filial piety. The new scale was developed with reference to the TDFPM that suggests that filial piety includes three dimensions; namely, the balance of interests, good affection, and family role norms (Wang and Zheng, 2015, pp. 272–273). The reliability and validity of the scale was then verified through empirical analysis using data from four investigations of Chinese working adults.

The three-factor structure of filial piety obtained using the EFA was in line with the TDFPM. Furthermore, it was found that GA was a stronger explanatory factor than FRN or BI in explaining filial piety. This was based on the finding that, compared with the FNR and BI, the GA had the highest (a) eigenvalue (GA: 5.427; FRN: 1.717; BI: 1.231), (b) percent variance (GA: 36.1795%; FRN: 11.448%; BI: 8.204%), (c) factor total score (GA: 27.25; FRN: 23.70; BI: 24.88), and (d) item average (GA: 5.39–5.53; FRN: 4.87–4.92; BI: 4.69–5.21). These results indicated that the GA made the highest contribution to the total variance (results a and b), and participants had the highest agreement with GA items (results c and d). Hence, it can be concluded that Chinese working adults, growing up in Confucian culture and influenced by Western individualism, still attach great importance to the good affection aspect of filial piety. This coincides with Yeh’s (2009) viewpoint that the basic emotion of filial piety is based on the good nature of human beings.

The CFA was used in this study to investigate and confirm the three-factor structure. The findings revealed that the three-factor model provided a good fit to the data. The reliability of the scale, evaluated by computing *R*^2^, AVE, Cronbach’s alpha coefficient, and split-half reliability, showed that the TDFPS had good cross-item stability. Furthermore, the test–retest reliability results indicated that the scale had good cross-time stability. The validation analyses showed that the scale had good psychometric properties and adequate concurrent and discriminant validity.

The results of the measurement invariance showed excellent configural, metric, and scalar measurement equivalence, which affirmed that the three-factor structure applies equally well to different genders, age groups, and cohabitation situations. Many studies have focused on gender and age differences in filial piety (Matthews, 1995; Chappell and Kusch, 2007), but little attention has been paid to the cohabitation situations between children and their parents. However, in fact, filial piety, especially filial behavior, is vulnerable to the distance between children and their parents. In contemporary China, with an aggravation of inter-regional population flow, more and more people no longer stick to the old motto of “when parents are alive, children should not travel too far afield” and live in different cities than their parents. They regard it as an important act of filial piety to often take their spouse and children home to visit their parents (Wang et al., 2014). However, this is not necessary for children living in the same house with their parents. People who live with their parents have difficulty and may make conjectures when they answer questions such as “I visit my parents regularly if I am not living with them” (Lum et al., 2015). Therefore, the characteristics of filial piety under the three main living situations (i.e., live in the same house, live in the same city/town but in a different house, and live in a different city/town) were fully considered to ensure the measurement invariance of the scale. Thus, the CFPS can be compared across different gender and age groups, as well as different cohabitation situations. These findings demonstrate the robustness of the three-factor structure of the TDFPS.

The DFPS and CFPS were used to evaluate criterion validity. The results of a correlation analysis were consistent with the expectations: The TDFPS was positively correlated with the DFPS and CFPS, with a low correlation coefficient. Furthermore, all three dimensions (FRN, BI, and GA) were positively correlated with RFP, PO, and CR, while BI was negatively correlated with AFP. This indicated that the DFPS has good criterion validity, and that the TDFPS is not a mechanical repetition of the DFPS or CFPS, but rather a further expansion of the filial piety measurement based on previous studies. Both the RFP and CR emphasize emotional caregiving. The GA also includes a measurement of emotional motivation, which can distinguish whether children are filial to their parents out of sincerity. Consistent with Lum et al. (2015), it is believed that the authoritative filial piety in the DFPS is no longer the main content of contemporary filial piety, so it was excluded from the TDFPS. However, as an important cultural tradition, the value of filial piety as a moral norm still exists. Therefore, the FRN dimension is proposed. Lum et al. (2015) emphasized intergenerational equality and filial piety within the scope of the children’s ability and embodied this idea in both the PO and CR. The present research builds upon that of Lum et al. (2015) and uses the BI dimension to measure the balance between rationality and morality in filial piety.

### Strengths and Limitations

In contrast to previous research on filial piety scales, this study has three main strengths. First, the new scale was developed using a combination of theory-driven and data-driven information, which is regarded as the best way to produce robust models and measures compared with using theory-driven or data-driven information alone (Jiang, 2004; Xiong et al., 2018). In this study, preliminary items based on the TDFPM were first generated and then revised them according to empirical results obtained from four samples of Chinese working adults. Second, the target group in this study was working adults, who are the primary source of support for the elderly in current Chinese society. Due to the influence of the traditional filial piety culture and the limitations of the current social security system in China, working children are the primary source to support the elderly. Whether they have filial piety consciousness and what kind of filial piety they hold will not only affect the success or failure of a family providing for the aged, but also affect the physical and mental health and quality of life of the elderly. Therefore, it is extremely important to explore the characteristics of filial piety in working children.

Third, the TDFPS can not only be used to compare individual levels of filial piety on a single dimension, like other filial piety scales, such as the DFPS and CFPS, but also it can be divided into eight types according to the three-dimensional hexapolar model. The TDFPS can measure the degree of preference for the polarity of each dimension. In this way, the subtypes in each dimension can first be measured and then synthesized with the subtypes of the three dimensions to obtain the overall filial piety type. According to different combinations of preferences in the three dimensions, filial piety can be divided into eight types as follows: true-autonomy-reasonable, true-autonomy-unreasonable, false-autonomy-reasonable, false-autonomy-unreasonable, true-heteronomy-reasonable, true-heteronomy-unreasonable, false-heteronomy-reaonable, and false-heteronomy-unreaonable. The three dimensions are shared by all individuals. However, people’s identification of each dimension is different. These eight types constituted by the three dimensions are supposed to have different distribution and influence in different populations, which needs to be explored in future studies.

Several limitations of this study are worth noting and may provide direction for further research. First, the proportion of middle-aged people over 40 years old in this study was relatively small (14.54% for EFA, 19.49% for CFA). These people belong to the “sandwich” generation and have elderly and children in their family to take care of. Some people even have to face conflicts between caring for their parents and their grandchildren. An examination of their understanding of the morality and rationality of filial piety and the balance of rights and interests is also valuable for research. Second, the TDFPS is a self-evaluation scale, and it can be difficult to gain reliable self-insight in a global tendency assessment. This can lead to memory bias and desirability-related distortions in participant responses (Kahneman et al., 2004; Schwarz et al., 2009; Brienza et al., 2018). This scale was completed anonymously. Participants were reminded of the confidentiality of the research results and asked to respond to the scale according to their actual situation. Although it is not possible to eliminate participant response biases, these procedures may reduce them to a certain degree (Xiong et al., 2018). To improve the ecological validity and reduce the social desirability biases, a state-level three-dimensional filial piety scale can be compiled by utilizing the event reconstruction method (ERM) proposed by Kahneman et al. (2004). The ERM provides greater access to episodic memory by illuminating details (Wagenaar, 1986; Robinson and Clore, 2002) and can vividly re-evoke certain events or episodes from the past (Grube et al., 2008). However, to achieve a stable filial piety measurement through the ERM, researchers need to use the scale for repeated measurements over a period of time, such as 1 month or 1 year. This increases the difficulty and cost of measurement.

## Conclusion

The two main conclusions of this study are as follows: (1) The TDFPM was supported, and the TDFPS duly included three dimensions; namely the balance of interests, good affection, and family role norms. (2) The newly developed TDFPS is a reliable and valid measurement of filial piety with good psychometric properties and can be used to measure filial piety across different age, gender, and cohabitation situations.

## Ethics Statement

This study was conducted following the approval by the Ethics Committee of the Psychological Experiment Teaching Center, Nanjing Normal University. All participants gave their informed consent in accordance with the Declaration of Helsinki. Participants were allowed to withdraw from the study whenever they wanted, and the data were collected anonymously.

## Author Contributions

JS contributed to the item generation, data collection, data analysis, and writing of the manuscript. FW contributed in providing theoretical guidance and writing of the manuscript.

## Conflict of Interest Statement

The authors declare that the research was conducted in the absence of any commercial or financial relationships that could be construed as a potential conflict of interest.
