# Supplementary material for: Three-Dimensional Filial Piety Scale: Development and Validation of Filial Piety Among Chinese Working Adults
Source: Front Psychol. 2019 Sep 6;10:2040. doi: 10.3389/fpsyg.2019.02040 (PMC6743377; doi:10.3389/fpsyg.2019.02040)
Supplement: Supplementary file 1 [file Table_1.docx]

**Appendix:**

In this survey, two questions needed to be answered for each item:

First, read the two sentences each item contains in the following table carefully, then choose the sentence that you identify with more with “√” in the left bracket.

Second, select the degree of identification with your choice represented by the Arabic numerals on the right side of the table (1 for slightly identify, 2 for moderately identify, 3 for completely identify) with “√”.

Please read the following sentences carefully, then make the best choice based on your situation. This questionnaire is completely anonymous and used only for scientific research. Please feel free to answer honestly since the results are completely confidential. Your answers are very important to us. Thank you very much.

| **No.** |  | **Item** | **Slightly identify** | **Moderately identify** | **Completely identify** | **Dimension** | **Item adapted from** |
| --- | --- | --- | --- | --- | --- | --- | --- |
| 1 | ( )  ( ) | I take initiative to accompany my parents if time permits.  *I passively accompany my parents only when they ask. | 1 | 2 | 3 | FRN | Written by first author |
| 2## | ( )  ( ) | *I seldom care about my parents’ inner feelings and think that giving them enough money is filial piety.  I care about my parents’ inner feelings rather than simply giving them money. | 1 | 2 | 3 | GA | Written by first author |
| 3## | ( )  ( ) | I only follow my parents’ reasonable advice.  * Whether my parents’ advice is appropriate or not, I strictly follow it. | 1 | 2 | 3 | BI | Written by first author |
| 4## | ( )  ( ) | When providing help to parents, they can feel my respect for them.  * When providing help to parents, they can feel the burden of their child. | 1 | 2 | 3 | GA | Written by first author |
| 5## | ( )  ( ) | When my parents have no financial capacity, I offer to support them.  * When my parents have no financial capacity, I support them only at the request of others. | 1 | 2 | 3 | FRN | CFPS #2 (Lum et al., 2015) |
| 6## | ( )  ( ) | * The reason why I accompany my parents for the Spring Festival is to avoid the sense of loss caused by not being able to reunite.  As long as the conditions permit, I must accompany my parents to celebrate the Spring Festival in order to satisfy their desire for reunion. | 1 | 2 | 3 | GA | Written by first author |
| 7## | ( )  ( ) | I take the initiative to offer sacrifices to my deceased parents.  * At the request of the family, I offer sacrifices to my deceased parents. | 1 | 2 | 3 | FRN | Written by first author |
| 8## | ( )  ( ) | When I help my parents, I am happy with them.  * When I help my parents, I have a tough attitude toward them. | 1 | 2 | 3 | GA | Written by first author |
| 9 | ( )  ( ) | *I am passively concerned about my parents’ health conditions when reminded by other family members.  I actively care about my parents’ health conditions in peacetime. | 1 | 2 | 3 | FRN | DFPS #1 (Yeh & Bedford, 2003) |
| 10## | ( )  ( ) | * In order to let my parents share housework, I would like to live with them.  In order to take better care of my parents, I would like to live with them. | 1 | 2 | 3 | GA | Written by first author |
| 11 | ( )  ( ) | I refuse resolutely if my parents ask me to take advantage of work to get some convenience that goes against the principles.  *I obey totally if my parents ask me to take advantage of work to get some convenience that goes against the principles. | 1 | 2 | 3 | BI | Written by first author |
| 12## | ( )  ( ) | * When my parents need to be taken care of and I can't satisfy them well, I don’t feel guilty as long as I am not criticized by others.  I feel guilty when my parents need to be taken care of but I can’t satisfy them well. | 1 | 2 | 3 | FRN | Written by first author |
| 13 | ( )  ( ) | I will remind my parents that arguing with others for queue jumping is wrong.  *I will do my best to defend my parents if they argue with others for queue jumping. | 1 | 2 | 3 | BI | Written by first author |
| 14 | ( )  ( ) | *I visit my parents passively only when they ask me to on holidays.  I take initiative to visit my parents on holidays. | 1 | 2 | 3 | FRN | Written by first author |
| 15 | ( )  ( ) | Rational suggestions from my parents will be adopted in my future programming.  *All suggestions, whether rational or not, from my parents will be adopted in my future programming. | 1 | 2 | 3 | BI | CFPS #7 (Lum et al., 2015) |
| 16 | ( )  ( ) | Showing filial piety to my parents is a natural expression of my real feelings.  *Showing filial piety to my parents is just an act for others. | 1 | 2 | 3 | GA | Written by first author |
| 17## | ( )  ( ) | * At the reminder of my family, I remember my parents birthdays.  I keep my parents’ birthdays firmly in mind. | 1 | 2 | 3 | FRN | Written by first author |
| 18 | ( )  ( ) | I will only support the right side if my spouse and parents disagree with each other.  *I will completely support my parents if my spouse and parents disagree with each other, even if my spouse is right. | 1 | 2 | 3 | BI | FBS #18 (Chen, Ho, & Tang, 2007) |
| 19## | ( )  ( ) | * I only do housework at the request of my parents.  As long as I have time, I take the initiative to undertake housework. | 1 | 2 | 3 | FRN | Written by first author |
| 20## | ( )  ( ) | When offering sacrifices to dead parents or ancestors, the true feelings are more important than the sacrificial rites.  * When offering sacrifices to dead parents or ancestors, sacrificial rites are the most important. | 1 | 2 | 3 | GA | Written by first author |
| 21## | ( )  ( ) | I take care of my parents when they are sick.  * I take care of my sick parents only at the request of the family. | 1 | 2 | 3 | FRN | CFPS #3 (Lum et al., 2015) |
| 22## | ( )  ( ) | * When my parents blame me for nothing, I usually put up with it in silence.  When my parents blame me for nothing, I will refute it appropriately. | 1 | 2 | 3 | BI | Written by first author |
| 23## | ( )  ( ) | I do my best to satisfy my parents’ reasonable wishes.  * Whether my parents’ wishes are reasonable or not, I will try my best to satisfy them. | 1 | 2 | 3 | BI | CFPS #9 (Lum et al., 2015) |
| 24 | ( )  ( ) | *I will talk to my parents at home only when they ask.  I will take the initiative to talk to my parents at home. | 1 | 2 | 3 | FRN | FBS #17(Chen, Ho, & Tang, 2007) |
| 25## | ( )  ( ) | When my parents do something that damages my legitimate rights and interests, I will resist appropriately.  * Even if my parents do something that damages my legitimate rights and interests, I will never resist. | 1 | 2 | 3 | BI | Written by first author |
| 26## | ( )  ( ) | When I learn that my parents are short of money, I take the initiative to provide financial assistance to them.  * Only at the request of my parents can I provide financial assistance to them. | 1 | 2 | 3 | FRN | Written by first author |
| 27 | ( )  ( ) | *Inheriting my parents’ legacy is the reason I take care of them.  Making my parents live more comfortably is the reason I take care of them. | 1 | 2 | 3 | GA | DFPS #9 (Yeh & Bedford, 2003) |
| 28## | ( )  ( ) | * I am willing to pay any price for filial piety to my parents.  I show filial piety to my parents within my ability. | 1 | 2 | 3 | BI | Written by first author |
| 29 | ( )  ( ) | Since I love my parents, I do my best to make them happy.  *Benefiting more from my parents is the reason I strive to make them happy. | 1 | 2 | 3 | GA | Written by first author |
| 30## | ( )  ( ) | * As long as my parents’ ideas are good for themselves, I will support them even if they hurt others.  When my parents’ ideas may hurt others, I advise them to give up properly. | 1 | 2 | 3 | BI | Written by first author |
| 31## | ( )  ( ) | When it comes to filial piety toward parents, emotion is more important than form.  * When it comes to filial piety toward parents, form is more important than emotion. | 1 | 2 | 3 | GA | Written by first author |
| 32 | ( )  ( ) | I will never violate the rights or interests of others when I show filial piety to my parents.  *I will do my best to take care of my parents even if I violate the rights and interests of others. | 1 | 2 | 3 | BI | Written by first author |
| 33## | ( )  ( ) | * When educating children, I let my parents do whatever they want, even if they are wrong.  When educating children, I will confront my parents if I find that they are not doing it right. | 1 | 2 | 3 | BI | Written by first author |
| 34 | ( )  ( ) | *Fear of being criticized by others is the reason I take care of my parents.  Gratitude for their fostering is the reason I take care of my parents. | 1 | 2 | 3 | GA | CFPS #6 (Lum et al., 2015) |
| 35 | ( )  ( ) | I often show care for my parents through calling them when I am far away.  *I show care for my parents only when they call me when I am far away. | 1 | 2 | 3 | FRN | FBS #15 (Chen, Ho, & Tang, 2007) |
| 36 | ( )  ( ) | I enjoy spending time accompanying my parents to make them happy.  *Accompanying my parents, for me, is just done to create the image of a filial son/daughter. | 1 | 2 | 3 | GA | Written by first author |
| 37# | ( )  ( ) | Promoting my parents’ happiness is one of my life plans.  I don’t actively take the happiness of parents as part of my life plan | 1 | 2 | 3 | FRN | Written by first author |
| 38# | ( )  ( ) | I take the initiative to take my parents for physical examinations regularly.  I take my parents for physical examinations at the request of my parents or others. | 1 | 2 | 3 | FRN | Written by first author |
| 39# | ( )  ( ) | On the issue of children’s education, I will listen to my parents, even if their ideas are rigid and incorrect.  On the issue of children’s education, I think parents’ opinions are unscientific and stick to my own way. | 1 | 2 | 3 | BI | Written by first author |
| 40# | ( )  ( ) | In order to realize the desire of parents to stay with their children, I am willing to give up my ideal job.  I stick to my favorite job and usually take care of my parents’ mental and physical health. | 1 | 2 | 3 | BI | Written by first author |
| 41# | ( )  ( ) | I take photos of my parents in order to retain their good moments.  I take photos of my parents in order to update personal social media accounts such as WeChat. | 1 | 2 | 3 | GA | Written by first author |
| 42# | ( )  ( ) | Filial piety is the responsibility of being a child and has nothing to do with others’ evaluation.  Filial piety can leave a good impression on people, so we should actively carry it out. | 1 | 2 | 3 | GA | Written by first author |

The serial number in the column “No.” is the same as that of the EFA. * means negative content in each dimension, that is, unreasonable, false, or heteronomy filial piety.

# Means this item was later discarded by the expert or target group assessment and not included in the EFA.

## Means this item was later discarded in the EFA and not included in the CFA.

DFPS, Dual Filial Piety Scale; CFPS, Contemporary Filial Piety Scale; FBS, Filial Behavior Scale.
